# Agri-vision Bangladesh: A multi-crop augmented image dataset for automated disease diagnosis in Bottle Gourd, Zucchini, Papaya, and Tomato

**DOI:** 10.1016/j.dib.2026.112528

**Published:** 2026-01-29

**Authors:** Md Masum Billah, Md. Anisur Rahman, Saifuddin Sagor, Sanzida Parvin, Mohammad Shorif Uddin

**Affiliations:** a Department of Computer Science and Engineering, Daffodil International University, Dhaka, 1216, Bangladesh; b Department of Computer Science & Engineering, Jahangirnagar University, Savar, Dhaka, 1316, Bangladesh

**Keywords:** Artificial intelligence, Computer vision, Data science, Machine learning, Bottle gourd leaves, Papaya Leaves, Zucchini Leaves, Tomato Leaves

## Abstract

This article introduces Agri-Vision Bangladesh, a comprehensive, augmented image dataset designed to advance automated disease diagnosis in four economically vital agricultural crops: Bottle Gourd (*Lagenaria siceraria*), Zucchini (*Cucurbita pepo*), Papaya (Carica papaya), and Tomato (*Solanum lycopersicum*). Addressing the scarcity of region-specific agricultural data, a total of 5266 original images were acquired directly from diverse agricultural fields in Bangladesh using a SONY ALPHA 7 II full-frame camera under natural lighting conditions. The dataset encompasses 28 distinct classes, covering a wide spectrum of biotic stressors including viral (Mosaic Virus, Leaf Curl), fungal (Downy Mildew, Anthracnose, Alternaria Blight), bacterial (Bacterial Blight, Xanthomonas), and pest-induced damage (Insect Hole, White Spot), alongside Healthy samples. To ensure scientific reliability, each image underwent a rigorous two-stage validation process by senior agronomists. To tackle class imbalance and facilitate the training of data-intensive Deep Learning models, the dataset was expanded using a Python-based augmentation pipeline incorporating geometric transformations (rotation, flipping) and photometric adjustments (noise, brightness) resulting in a final repository of 28,000 images (5266 original and 22,734 augmented). All files are standardized to 512×512 pixels in JPG format. This expert-validated resource serves as a critical benchmark for developing robust computer vision algorithms (e.g., CNNs, Vision Transformers) for precision agriculture, enabling research into fine-grained classification, object detection, and cross-crop transfer learning in subtropical farming environments.

Specifications Table

**Table:** 

Subject	Computer Sciences
Specific subject area	Artificial Intelligence, Computer Vision, Data Science, Machine Learning, Bottle-Gourd Leaves, Papaya Leaves, Zucchini Leaves, Tomato Leaves.
Type of data	Image
Data collection	We have compiled an extensive combined leaf disease dataset comprising 5266 high-quality images of Bottle Gourd, Zucchini, Papaya, and Tomato leaves. These are classified into 28 distinct categories, including 8 classes for Bottle-Gourd, 9 for Zucchini, 6 for Papaya, and 5 for Tomato, covering a broad spectrum of disease symptoms and healthy conditions. All images were captured under natural field conditions using a SONY ALPHA 7 II camera between January 13, 2024, and October 22, 2024, across Bangladesh, ensuring diverse environmental representation and providing a rich, reliable foundation for deep learning-based leaf disease analysis.
Data source location	Town/City/Region: Daffodil Smart City Country: Bangladesh
Data accessibility	Repository name: Mendeley Data Data identification number: 10.17632/8t6k37ztxc.2 Direct URL to data: https://data.mendeley.com/preview/8t6k37ztxc?a=a88a48f1-a9b0-4354-a081-cc8f1e936364
Related research article	None

## 1 Value of the Data

• This comprehensive repository integrates multi-crop imagery of Bottle Gourd, Zucchini, Papaya, and Tomato, addressing the critical scarcity of open-access data for high-value vegetable crops in humid subtropical regions. By covering 28 distinct pathological conditions, it enables the development of automated surveillance systems crucial for safeguarding yield and ensuring food security in developing agricultural economies.
• The dataset captures subtle morphological variations, and symptom overlaps among fungal, viral, and bacterial infections (e.g., distinguishing between visually similar leaf blights or crop-specific mosaic virus patterns). This fine-grained visual detail is essential for training deep convolutional neural networks (CNNs) and Vision Transformers to overcome the challenge of inter-class visual similarity, thereby reducing false-positive rates in automated diagnosis.
• Captured exclusively in uncontrolled field environments, the images preserve natural heterogeneity, including variable luminosity, complex background clutter, shadows, and occlusions. This ``in-the-wild'' characteristic validates the dataset's utility for developing robust computer vision models capable of generalizing well in practical deployment scenarios, such as edge-computing devices or mobile-based advisory tools for farmers.
• By providing a structured bifurcation between 5266 original expert-validated samples and 22,734 algorithmically augmented counterparts, this resource serves as a standard benchmark for evaluating the efficacy of synthetic data generation and augmentation strategies. It facilitates research into addressing class imbalance and data scarcity, particularly for rare biotic stressors where large-scale data collection is traditionally challenging.
• The multi-species nature of the dataset supports advanced research in domain adaptation and transfer learning, allowing models trained on these specific crops to potentially generalize features to other botanically related species. This capability is vital for creating scalable, crop-agnostic diagnostic tools within the broader precision agriculture ecosystem.

## 2 Background

The sustainable production of Bottle G`ourd, Zucchini, Papaya, and Tomato is critical for nutritional security in subtropical regions, yet it is severely challenged by biotic stressors. Pathological studies have characterized devastating outbreaks of Leaf Curl in Bottle Gourd [1,2], Mosaic Virus complexes in Zucchini [8,10], and fungal/viral infections in Papaya [5,7]. While molecular characterization [9,11] offers precise diagnosis, these methods are resource-intensive and impractical for large-scale field surveillance. Conversely, Computer Vision offers a scalable solution. Recent studies utilizing Deep Ensemble learning [4], Hybrid forecasting models [3], and automated Tomato disease detection [12] has demonstrated high diagnostic accuracy. Furthermore, advanced architectures like Capsule Networks [15] and Swin Transformers [16] show promise. However, the robustness of such models is currently limited by the scarcity of diverse training data, as existing datasets are often crop-specific [5,6] or lack the ``in-the-wild'' environmental heterogeneity required for real-world deployment [13,14]. This dataset addresses this gap by providing 28,000 expert-validated images across four crops, capturing the complex background clutter and variable lighting necessary to train generalized, field-ready AI systems.

## 3 Data Description

The Agri-Vision dataset represents a comprehensive collection of leaf imagery covering four economically significant crops: Tomato, Papaya, Zucchini, and Bottle Gourd. The dataset comprises a total of 28,000 images stored in JPG format, with all files standardized to a resolution of 512 × 512 pixels. The images were acquired between January 13, 2024, and October 22, 2024, primarily from agricultural fields in Daffodil Smart City (23.8769° N, 90.3113° E) and surrounding regions in Bangladesh.

The repository is organized into two primary parent directories:

• Original_Images: This directory contains 5266 raw, expert-validated images. It is subdivided into four crop-specific folders, which are further categorized into 28 distinct classes representing various disease symptoms and healthy conditions. The filenames in this directory follow the format Class Name Original Index.jpg (e.g., Tomato_Downy_001.jpg).
• Augmented_Images: To address class imbalance and enhance model robustness, this directory contains 22,734 augmented images. These samples were generated using geometric and photometric transformations derived from the original dataset. The augmented files are stored in corresponding class folders.

**Fig. 1:**
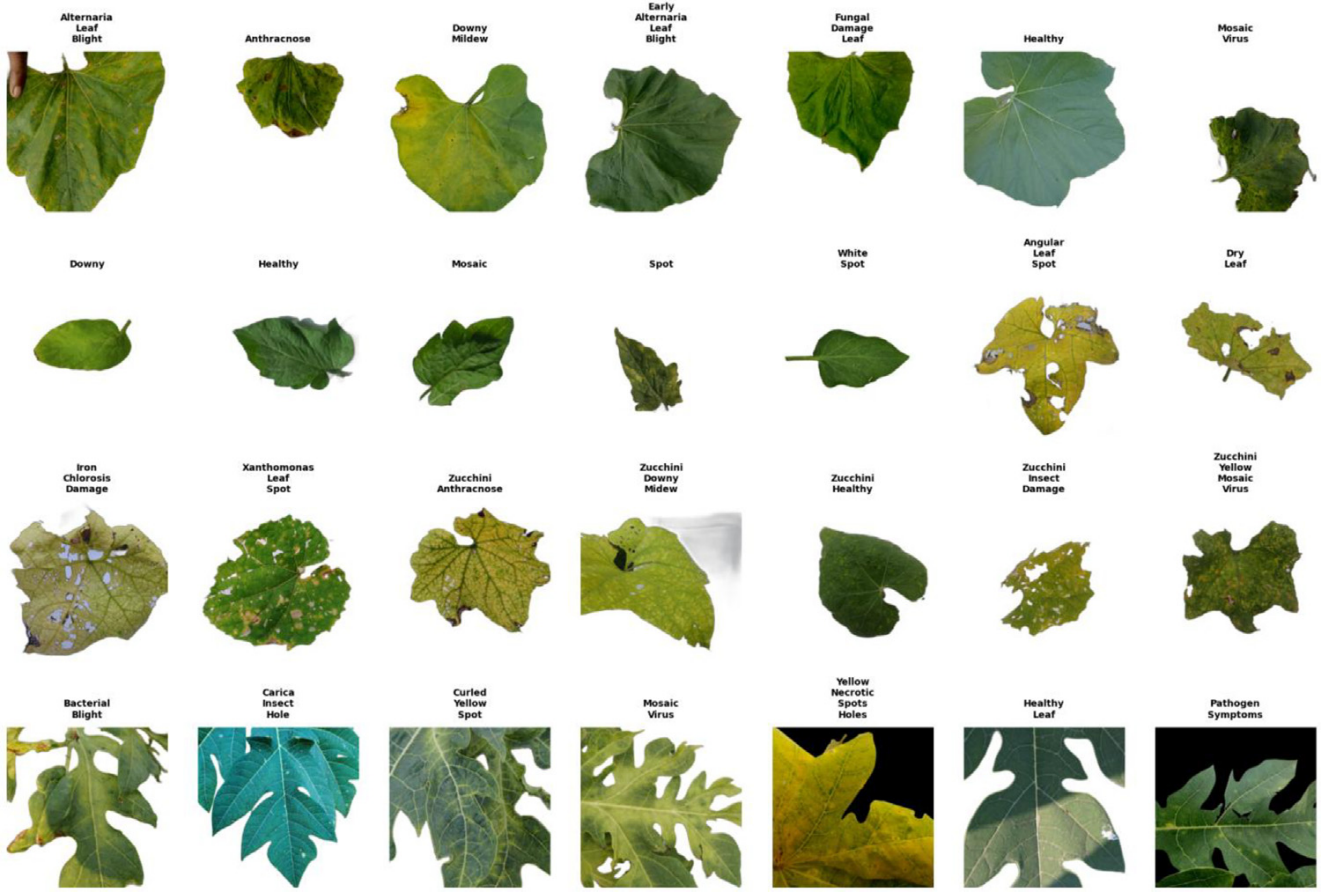
Representative sample images from the Agri-Vision dataset displaying various disease symptoms and healthy conditions for (a) Tomato, (b) Papaya, (c) Zucchini, and (d) Bottle Gourd leaves.

Table 1 provides a detailed statistical breakdown, listing the specific image counts for each class across the four crops along with a summary of visual symptoms. Table 2 offers an in-depth scientific description of the diagnostic characteristics for each disease class to aid in accurate identification. Additionally, Fig. 1 illustrates representative sample images from the dataset, showcasing the variability in disease manifestation, leaf morphology, and background complexity.

**Table 1:** Distribution of original and augmented images across 28 classes of Tomato, Papaya, Zucchini, and Bottle Gourd with a summary of visual symptoms.

Dataset	Class Name	Visual Symptoms (Summary)	Before (Original Images)	After Augmented Images
Tomato Leaf Diseases	Tomato Downy Mildew	Pale yellow chlorotic patches; tissue thinning.	57	943
	Tomato Healthy	Vibrant green, serrated margins; no lesions.	288	712
	Tomato Mosaic	Severe twisting, blistering, and mottling.	195	805
	Tomato Spot	Irregular dark necrotic lesions with halos.	311	689
	Tomato White Spot	Irregular white perforations/holes.	65	935
Papaya Leaf Diseases	Papaya Bacterial Blight	Water-soaked lesions; marginal necrosis.	183	817
	Papaya Carica Insect Hole	Irregular chewed holes; skeletonization.	318	682
	Papaya Curled Yellow Spot	Downward curling; rugose texture.	538	462
	Papaya Healthy Leaf	Deep green, palmately lobed; glabrous.	189	811
	Papaya Mosaic Virus	Distinct mosaic pattern; vein clearing.	119	881
	Papaya Pathogen Symptoms	Interveinal chlorosis; yellow speckling.	286	714
	Papaya Yellow Necrotic Spots Holes	Extensive yellowing; shot-holes.	51	949
Zucchini Leaf Diseases	Zucchini Angular Leaf Spot	Water-soaked; angular shot-holes.	120	880
	Zucchini Anthracnose	Dark lesions coalescing into patches.	129	871
	Zucchini Downy Zucchini Mildew	Angular yellow mosaic-like patches.	153	847
	Zucchini Dry Leaf	Senescent, brown, papery texture.	67	933
	Zucchini Healthy	Uniform green; hispid (rough) surface.	108	892
	Zucchini Insect Damage	Ragged holes without chlorotic halos.	78	922
	Zucchini Iron Chlorosis Damage	Interveinal yellowing (net-like pattern).	65	935
	Zucchini Xanthomonas Leaf Spot	Necrotic lesions with yellow halos.	86	914
	Zucchini Yellow Mosaic Virus	Severe blistering and deformation.	202	798
Bottle Gourd Leaf Diseases	Bottle Gourd Alternaria Leaf Blight	Concentric rings (target-board pattern).	303	697
	Bottle Gourd Anthracnose	Dark irregular necrotic patches.	276	724
	Bottle Gourd Downy Mildew	Marginal necrosis; yellow patches.	286	714
	Bottle Gourd Early Alternaria Leaf Blight	Tiny scattered brown spots.	179	821
	Bottle Gourd Fungal Damage Leaf	Scattered lesions; uneven texture.	39	961
	Bottle Gourd Healthy	Cordate shape; soft velvety texture.	260	740
	Bottle Gourd Mosaic Virus	Crinkling, twisting, and mottling.	315	685
Total	28		5266	22,734

**Table 2:** Morphological characterization and visual diagnostic criteria used for the annotation of disease classes across Bottle Gourd, Zucchini, Papaya, and Tomato.

Class	Description	Visualization
Tomato Downy Mildew	Irregular, pale yellow to light green chlorotic patches appear on the upper leaf surface, often originating from the margins. These ill-defined lesions lack distinct necrotic centers initially. The leaflet retains its lobed structure but exhibits mild tissue thinning and slight curling in affected zones.	
Tomato Healthy	The leaflet displays a uniform, vibrant deep green color with a matte texture and distinct pinnate venation. Its deeply lobed, serrated margins are intact. The surface is free from necrosis, chlorosis, lesions, or insect damage, representing a physiologically healthy specimen	
Tomato Mosaic	The leaflet displays a uniform, vibrant deep green color with a matte texture and distinct pinnate venation. Its deeply lobed, serrated margins are intact. The surface is free from necrosis, chlorosis, lesions, or insect damage, representing a physiologically healthy specimen.	
Tomato Spot	The leaflet displays large, irregular necrotic lesions, varying from dark brown to black, primarily originating at the leaf margins and tips. These lesions are frequently bordered by diffuse chlorotic (yellow) halos, indicating tissue degradation. As the condition advances, the affected areas become desiccated and papery, causing the leaf to curl inward or deform due to loss of structural integrity.	
Tomato White Spot	The leaflet exhibits distinct irregular white to translucent patches and significant tissue loss, characteristic of insect feeding or mechanical damage. These lesions manifest as perforations (holes) or areas of skeletonization, where the green mesophyll tissue has been removed, leaving a thin, membranous surface. The margins of these voids often show narrow necrotic browning, while the remaining laminar tissue largely retains its green pigmentation, distinguishing it from systemic chlorotic diseases.	
Papaya Bacterial Blight	The leaf exhibits irregular, water-soaked lesions that frequently originate at the leaf margins and tips. As the infection progresses, these areas turn dark brown to necrotic and brittle, often separated from the healthy green tissue by a diffuse chlorotic (yellow) zone. The necrotic tissue may eventually dry out and crack, giving the leaf a ragged appearance while the lesions expand inward between the veins.	
Papaya Carica Insect Hole	The leaf blade exhibits distinct physical damage characterized by irregular perforations and chewed margins, resulting from pest feeding activity. Unlike pathogen-induced lesions, these voids lack water-soaked halos or concentric rings. The damage patterns include complete tissue removal (holes) and areas of skeletonization or surface scraping, where the mesophyll is consumed, leaving a translucent membrane. While the structural integrity is compromised, the remaining non-damaged tissue largely retains its natural green pigmentation.	
Papaya Curled Yellow Spot	The foliage displays severe morphological distortion, primarily defined by downward curling and twisting of the leaf margins. The laminar surface exhibits significant rugosity (wrinkling) and a distinct mosaic-like pattern, where chlorotic (yellow) patches intermingle with dark green tissue. In advanced stages, the leaf lobes appear narrowed and thickened, while the veins may show signs of clearing or yellowing, indicative of viral infection stress.	
Papaya Healthy Leaf	The leaf exhibits a characteristic large, palmately lobed structure with deep incisions separating the lobes. The surface displays a uniform, vibrant deep green pigmentation, indicating optimal chlorophyll content and physiological health. A prominent network of pale-yellow to light-green veins radiates from the petiole attachment, creating a distinct reticulate pattern. The texture appears smooth (glabrous) and leathery, with absolutely no signs of chlorosis, necrosis, lesions, or insect damage.	
Papaya Mosaic Virus	The leaf blade displays a distinct mosaic pattern characterized by irregular, alternating patches of dark green and chlorotic (yellow-green) tissue. This mottling effect is frequently accompanied by vein clearing, where the vascular network appears translucent or lighter than the surrounding lamina. The surface texture often exhibits puckering or blistering, and the leaf margins may show mild distortion or curling as the viral infection disrupts cellular growth.	
Papaya Pathogen Symptoms	The leaf blade displays widespread interveinal chlorosis, manifesting as numerous small, scattered yellow flecks or spots (speckling) across the lamina. Unlike severe mosaic or leaf curl, the leaf structure remains relatively intact with minimal marginal distortion. The symptoms present as a faint mottling or stippling pattern where chlorophyll loss is localized, typically indicating an early-stage infection or mild pathogenic stress prior to the development of necrotic lesions.	
Papaya Yellow Necrotic Spots Holes	The foliage exhibits extensive chlorosis, where the majority of the leaf surface turns a vibrant yellow, indicating severe chlorophyll degradation. This discolored tissue is punctuated by numerous small, scattered necrotic specks (dark brown spots). Furthermore, the leaf blade is compromised by irregular holes and marginal tearing. These voids suggest that the necrotic tissue has desiccated and detached (shot-hole effect) or that the leaf has suffered structural disintegration due to the combined effects of advanced infection and tissue senescence.	
Zucchini Angular Leaf Spot	The leaf blade is characterized by numerous small, angular lesions that are strictly delimited by the leaf veins, giving them a geometric shape. In these specimens, the central necrotic tissue has desiccated and detached, resulting in a distinctive 'shot-hole' appearance with jagged perforations. The surrounding laminar tissue exhibits widespread chlorosis (yellowing), while the margins of the voids often retain a thin, dark brown necrotic border typical of bacterial infection.	
Zucchini Anthracnose	The foliage displays characteristic circular to irregular necrotic lesions, initially appearing water-soaked before turning dark brown or black. As the fungal infection advances, these spots frequently coalesce (merge together) to form expansive dead patches, particularly along the leaf veins and margins. The necrotic tissue becomes dry and brittle, leading to cracking or shredding of the leaf blade, while the surrounding green tissue may show signs of chlorosis due to stress.	
Zucchini Downy Mildew	The upper leaf surface exhibits distinctive angular chlorotic patches that are strictly delimited by the leaf veins, creating a blocky, mosaic-like appearance of yellow and green tissue. Unlike bacterial lesions, these areas primarily manifest as bright pale-yellow zones rather than water-soaked spots. In the advanced stages observed here, the chlorotic tissue has become necrotic (brown) and brittle, occasionally tearing or detaching to form irregular voids as the leaf undergoes senescence.	
Zucchini Dry Leaf	The leaf presents a generalized state of senescence and desiccation, characterized by a dull, pale yellow to brown discoloration across the entire surface. The tissue texture appears papery and brittle, lacking the turgidity and glossy finish of healthy foliage. Due to the loss of structural integrity, the leaf blade exhibits irregular tearing, ragged margins, and enlarged voids, often leaving only the venation framework intact in severely degraded areas.	
Zucchini Healthy	The leaf displays a broad, palmately lobed morphology with a cordate (heart-shaped) base and serrated margins. The surface exhibits a uniform, vibrant green coloration, indicative of active photosynthesis and optimal physiological health. The texture is characteristically hispid (rough), and the palmate venation network is distinct and intact. There are absolutely no visible signs of biotic stress, such as lesions, chlorosis, mosaic patterns, or insect damage.	
Zucchini Insect Damage	The leaf blade exhibits evident physical damage caused by pest feeding, manifesting as irregular perforations and ragged, chewed margins. Unlike bacterial or fungal lesions, these voids typically lack chlorotic halos or water-soaked borders. In severe cases, the leaf displays signs of skeletonization, where the soft mesophyll tissue is consumed while the tougher venation network remains partially intact, significantly reducing the photosynthetic surface area.	
Zucchini Iron Chlorosis Damage	The leaf displays characteristic interveinal chlorosis, where the tissue between the veins turns pale yellow to whitish while the vascular network remains green. This creates a distinct, contrasting reticulate (net-like) pattern across the surface. Unlike pathogen-induced spots, this physiological disorder affects the laminar pigmentation broadly. In severe cases, the chlorotic tissue may develop necrotic brown patches and become brittle, leading to irregular holes and marginal scorching.	
Zucchini Xanthomonas Leaf Spot	The leaf is heavily impacted by angular, water-soaked lesions that are constrained by the leaf venation. As the infected tissue dies, it turns necrotic and detaches, creating a perforated 'shot-hole' effect across the blade. The remaining tissue surrounding the lesions frequently displays a diffuse chlorotic (yellow) halo, characteristic of bacterial phytotoxicity. The infection leads to significant loss of photosynthetic area and structural degradation.	
Zucchini Yellow Mosaic Virus	The leaf exhibits severe symptoms typical of viral infection, most notably distinct mosaic mottling with alternating patches of bright yellow and dark green tissue. The laminar surface is characterized by intense puckering, blistering, and rugosity, giving the leaf a bubbly or uneven texture. Additionally, the leaf blade shows significant morphological distortion, including curling, twisting, and stunting, as the virus disrupts normal cellular expansion and chlorophyll distribution.	
Bottle Gourd AlternariaLeaf Blight	The leaf surface is marked by distinct circular to oval necrotic lesions, ranging from dark brown to black. A key diagnostic feature is the presence of concentric rings within the larger spots, creating a characteristic 'target-board' pattern. These lesions are frequently surrounded by a diffuse chlorotic (yellow) halo, indicating fungal toxin activity. As the disease progresses, the spots may enlarge and coalesce, leading to extensive blighting and premature leaf senescence.	
Bottle Gourd Anthracnose	The leaf exhibits characteristic dark brown to black necrotic lesions, often originating as water-soaked spots. These lesions are typically irregular in shape and vary in size. A prominent feature is the tendency of spots to coalesce (merge), forming extensive blighted patches, particularly along the leaf margins and veins. The affected tissue becomes dry and may crack, while the surrounding green area often shows signs of chlorosis due to tissue degradation.	
Bottle Gourd Downy Mildew	The upper leaf surface displays prominent pale yellow to brown chlorotic patches, which often expand inward from the margins. While some lesions appear constrained by major veins, others merge to form irregular blighted zones. In humid conditions, the underside may show fungal growth, though the primary visual symptom here is the extensive marginal necrosis and desiccation, where the infected tissue turns dark brown and brittle, contrasting sharply with the remaining green lamina.	
Bottle Gourd Early Alternaria Leaf Blight	The leaf presents initial symptoms of infection, characterized by tiny, scattered brown to black lesions. At this early stage, the spots are relatively small and isolated, lacking the pronounced concentric rings seen in advanced blight. Some lesions may exhibit a faint chlorotic halo, indicating the onset of tissue degradation. The overall leaf structure remains largely intact, though minor tissue depression may be observed at the infection sites.	
Bottle Gourd Fungal Damage Leaf	The leaf exhibits non-specific symptoms of fungal colonization, characterized by scattered necrotic lesions and irregular surface discoloration. The infected areas appear slightly sunken or softened, often leading to localized tissue collapse and the formation of small perforations or voids. Unlike specific blights, the damage pattern is uneven, with the surrounding green tissue showing signs of physiological stress but retaining its vascular structure.	
Bottle Gourd Healthy	The leaf displays a broad, cordate (heart-shaped) to pentagonal morphology with a gently undulating margin. The surface exhibits a uniform, vibrant green pigmentation, signifying optimal chlorophyll content and physiological health. The texture is characteristically soft and pubescent (velvety), with a prominent palmate venation network radiating from the petiole. The leaf blade is free from any necrotic lesions, chlorosis, deformations, or pest damage, representing a vigorous vegetative state.	
Bottle Gourd Mosaic Virus	The foliage displays significant morphological distortion, including crinkling, twisting, and downward curling of the leaf margins. The laminar surface exhibits a rugose or blistered texture, accompanied by a subtle mosaic mottling of light and dark green patches. The viral infection disrupts normal tissue expansion, leading to stunted growth and irregular leaf shapes, while the venation may show signs of clearing or thickening in affected zones.

## 4 Experimental Design, Materials and Methods

### 4.1 Methodological workflow

**Fig. 2:**
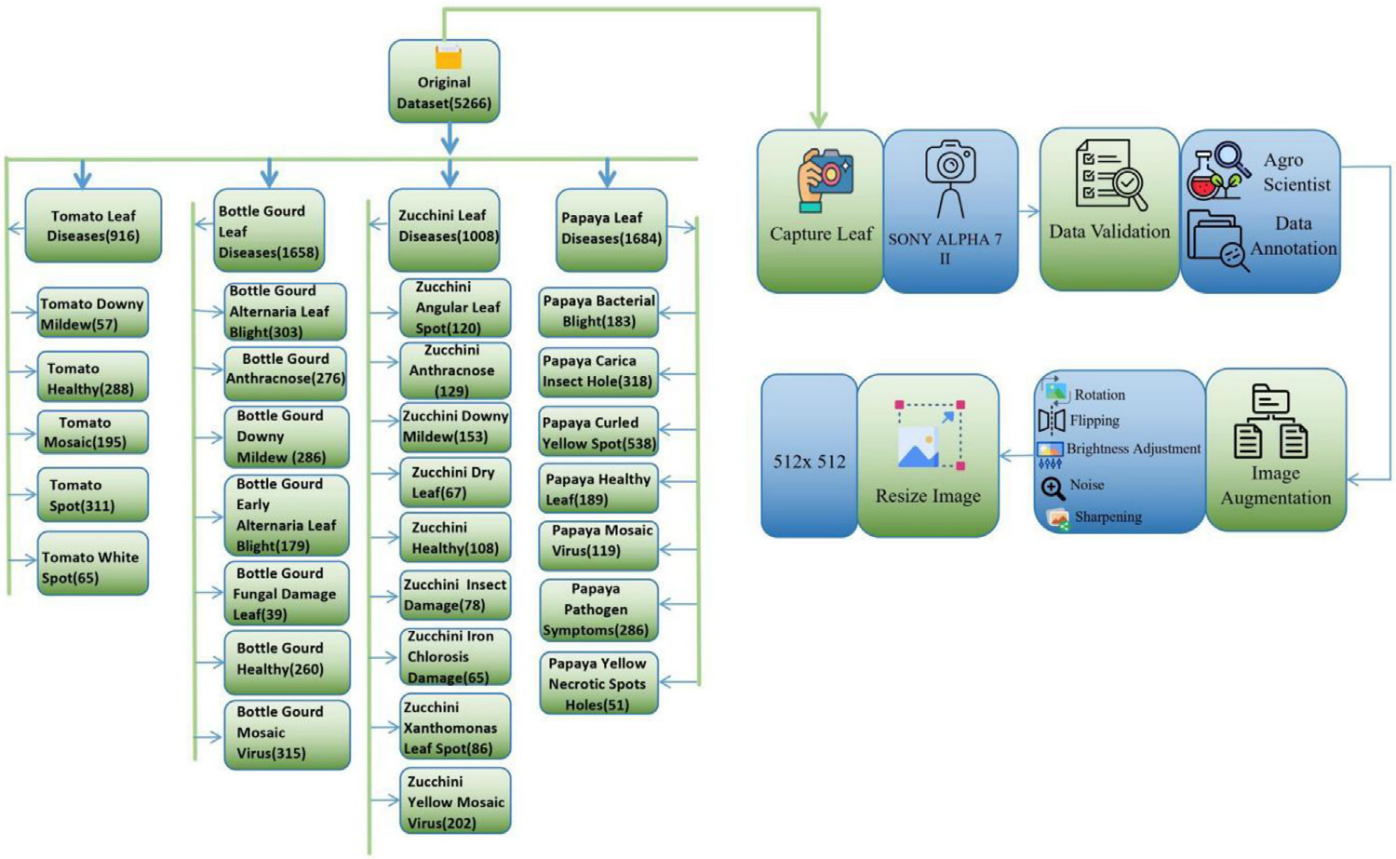
Schematic workflow of the dataset development process, illustrating image acquisition, expert validation, pre-processing, augmentation, and final folder organization for Bottle-Gourd, Zucchini, Papaya and tomato leaf diseases.

Fig. 2 Comprehensive workflow of the Agri-Vision Bangladesh dataset curation and hierarchical class distribution. The diagram is bifurcated into two functional segments: (Left Panel) A taxonomic tree. structure detailing the class-wise distribution of 5266 original expert-validated images across four crop categories (Tomato, Bottle Gourd, Zucchini, and Papaya). Specific sample counts for all 28 distinct disease classes and healthy conditions are explicitly enumerated to demonstrate dataset diversity. (Right Panel) The sequential data processing pipeline, commencing with in-situ image acquisition using a SONY ALPHA 7 II camera, followed by rigorous validation by agricultural scientists. The workflow highlights the standardization of all samples to a resolution of 512×512 pixels and the subsequent application of a Python-based augmentation module, incorporating geometric (Rotation, Flipping) and photometric (Brightness, Noise, Sharpening) transformations, to generate the final robust repository for deep learning applications.

### 4.2 Camera specification

For image collection, we used a SONY ALPHA 7 II mirrorless camera as our primary image-capturing device. Equipped with a 24.3 MP full-frame (35 mm) Exmor CMOS sensor, this camera provides high dynamic range and low-noise performance suitable for agricultural field photography. Images were captured using variable focal lengths to document different aspects of the diseases: 28 mm (Wide) for environmental context, 35 mm for balanced texture, 50 mm for standard depth, and 70 mm (Telephoto) for capturing fine lesion details without disturbing the foliage. To ensure image consistency, all samples were photographed under optimal natural lighting conditions (avoiding direct midday sun) to preserve true color representation and surface gradients. These measures minimized environmental distortion and ensured high-fidelity data suitable for machine learning analysis. The detailed specifications are presented in Table 3.

**Table 3:** Details of camera specification:.

Model Name	Shooting Mode	Resolution (MP)	Focal Length (mm)	Sensor Size
SONY ALPHA 7 II	Wide Angle	24.3	28	Full-frame (35 mm)
SONY ALPHA 7 II	Landscape	24.3	35	Full-frame (35 mm)
SONY ALPHA 7 II	Portrait	24.3	50	Full-frame (35 mm)
SONY ALPHA 7 II	Telephoto	24.3	70	Full-frame (35 mm)

### 4.3 Dataset collection and processing

The combined leaf disease dataset integrates high-quality images of Papaya, Zucchini, Bottle Gourd, and Tomato leaves, systematically collected to capture a wide range of disease symptoms and healthy conditions. All samples were photographed directly in agricultural fields across Bangladesh (specifically Savar, Ashulia, and Daffodil Smart City) between January 13, 2024, and October 22, 2024, using a SONY ALPHA 7 II camera under natural lighting. To ensure diagnostic accuracy, a rigorous validation process was implemented involving three senior agronomists from the Department of Agricultural Science at Daffodil International University. Each expert independently annotated the images based on visual symptoms, and in cases of disagreement, a majority voting consensus method was applied to determine the final class label.

Following validation, the dataset underwent standardization and augmentation to address class imbalance and enhance model robustness utilizing a custom script developed in Python with Albumentations and OpenCV libraries. The augmentation pipeline incorporated both photometric and geometric transformations. Specifically, geometric variations were introduced through random rotations (limit=±30°, *p* = 0.7) and horizontal (*p* = 0.5) and vertical (*p* = 0.2) flipping. Photometric adjustments involved RandomBrightnessContrast (limit=0.2, *p* = 0.5) and GaussNoise injection (var_limit=10.0–50.0, *p* = 0.2) to simulate realistic sensor variability. All original and augmented images were finally resized to 512×512 pixels and saved in JPG format, resulting in a total dataset of approximately 28,000 samples.

### 4.4 Comparison with existing datasets

Table 4 describes the comparison with the existing datasets

**Table 4:** Comparison with existing datasets.

Ref.	Name of Data	Size of dataset	Source of dataset
[4]	Deep Ensemble Framework for Bottle Gourd (Nuhash et al., 2025)	7000 images covering 7 classes (5 diseases + 2 healthy). Focuses on ensemble learning.	Collected in Bangladesh Limitation Focuses only on Bottle Gourd. Our Advantage: We integrate Bottle Gourd with Zucchini, Papaya, and Tomato for multi-crop modeling.
[5]	BDPapayaLeaf Dataset (Mustofa et al., 2025)	2159 original images categorized into 5 classes. Tailored for CNN and YOLO models.	A robust country-specific dataset. Limitation: Single-crop focus. Our Advantage: Our dataset includes Papaya along with three other crops, enabling broader generalization tests.
[12]	Automated Tomato Disease Detection (Ahmed et al., 2024)	60,000 images (mix of PlantVillage and field data) covering 10 distinct illness classes.	Large-scale dataset Limitation: Often relies on lab-controlled or pre-existing public data. Our Advantage: Our Tomato images are exclusively field-captured with complex backgrounds, ensuring "in-the-wild" robustness.
[3]	RF-ANN Forecasting for Bottle Gourd (Chittaragi et al., 2025)	Weather and severity data for Anthracnose forecasting.	Limitation: Focuses on numerical weather data, not image classification. Our Advantage: We provide visual data (images) which is essential for Computer Vision applications.
Our Work	Agri-Vision Bangladesh (Proposed Dataset)	28,000 Total Images (5266 Original + 22,734 Augmented) across 4 Crops (Bottle Gourd, Zucchini, Papaya, Tomato) covering 28 Classes.	Unique Contribution: Combines four major high-value crops in a single repository. Includes rare classes, verified by agronomists, and follows strict Data in Brief 2025 guidelines (min 50 originals/class).

## Limitations

While our work encompasses Bottle Gourd, Zucchini, Papaya, and Tomato leaf disease datasets, it is not without limitations. Although the combined dataset is extensive, a primary limitation is its geographical bias, as the images were acquired exclusively from agricultural fields in Bangladesh. Consequently, it may not capture every possible variation in leaf appearance, local soil backgrounds, or specific disease strains found across different global climates. Variations in lighting conditions, background clutter, and subtle disease symptom manifestations are representative of this specific subtropical environment, which may affect model generalization when applied to real-world scenarios in regions with significantly different environmental characteristics. Additionally, despite the use of augmentation to balance the classes, some rare disease types or early-stage infection symptoms might still be underrepresented compared to widely prevalent conditions. However, strictly regarding the scope of this study, all insights and recommendations presented here stem directly from our field experiments and aim to provide a solid baseline for advancing multi-crop disease detection.

## Ethics Statement

We confirm that the authors have read and followed the ethical requirements for publication in Data in Brief. The dataset was collected in collaboration with Daffodil International University and Jahangirnagar University, Bangladesh.

## Credit Author Statement

**Md Masum Billah:** Conceptualization, Methodology, Writing; **Md Anisur Rahman:** Data Curation, Methodology; **Saifuddin Sagor:** Data Acquisition, Formal analysis; **Sanzida Parvin:** Data Acquisition; **Mohammad Shorif Uddin:** Supervision, Writing – Review & Editing.

## Data Availability

Mendeley DataAgri-Vision4: A Comprehensive Multi-Crop Leaf Disease Dataset of Tomato, Papaya, Zucchini, and Bottle Gourd from Bangladesh (Original data)
